# Associations of Apolipoprotein ε4 Genotypes with Motor and Nonmotor Symptoms in Parkinson's Disease: A Cross‐Sectional Study

**DOI:** 10.1002/mdc3.13862

**Published:** 2023-08-25

**Authors:** Ali Kapan, Sandra Haider, Maria Wakolbinger, Josef Spatt

**Affiliations:** ^1^ Department of Social and Preventive Medicine, Center for Public Health Medical University of Vienna Vienna Austria; ^2^ Faculty for Medicine Sigmund Freud University Vienna Vienna Austria; ^3^ Neurological Department Evangelical Hospital Vienna Vienna Austria

**Keywords:** *APOE* ε4 allele, non‐motor symptoms, Parkinson's disease, cognitive decline

## Abstract

**Background:**

The apolipoprotein E (*APOE*) ε4 allele has been associated with cognitive decline in Parkinson's disease (PD), but little is known about its relationship with motor and other nonmotor symptoms and whether *APOE* ε4 retains an influence on cognition when other factors are considered.

**Objective:**

To investigate the impact of *APOE* ε4 on motor/nonmotor symptoms and its relationship with other factors affecting cognition in individuals with PD.

**Methods:**

We analyzed data from 7616 individuals, comparing motor/nonmotor symptoms in different *APOE* genotypes using binary logistic regression. Multivariate logistic regression examined factors associated with cognitive impairments, including *APOE* ε4, Geriatric Depression Scale (GDS) score, Non‐motor Symptom Questionnaire (NMS) score, Movement Disorder Society–Sponsored Revision of the Unified Parkinson's Disease Rating Scale (MDS‐UPDRS) Part II score, and physical activity level.

**Results:**

*APOE* ε4 heterozygosity was modestly associated with lower cognitive scores (odds ratio [OR], 0.92; 95% confidence interval [CI], 0.87–0.99), whereas no significant association was found for any other nonmotor and motor symptoms. However, in multivariate analysis, cognitive impairment was associated with higher GDS (OR, 1.28; 95% CI, 1.23–1.34), NMS (OR, 1.22; 95% CI, 1.19–1.25), and MDS‐UPDRS Part II (OR, 1.07; 95% CI, 1.06–1.09) scores, whereas physical activity was negatively associated (OR, 0.99; 95% CI, 0.98–0.99). *APOE* ε4 was no longer significant after adjusting for these factors.

**Conclusions:**

There is a link between cognition and *APOE* ε4 in patients with PD; however, when considering multiple factors, *APOE* ε4 plays a subordinate role. Other factors, such as depression, physical activity, and other nonmotor symptoms, demonstrate a stronger influence on cognitive impairment.

Genome‐wide association studies have identified several genetic susceptibility loci for Parkinson's disease (PD), including the apolipoprotein E (*APOE*) ε4 allele.^1^ The *APOE* ε4 allele is a significant genetic risk factor for sporadic Alzheimer's disease (AD) and dementia with Lewy bodies.^2^, ^3^ Several studies have reported an association between *APOE* ε4 status and cognitive impairment in individuals with PD, indicating its potential role as a genetic risk factor for the development of cognitive decline in these patients.^4^, ^5^, ^6^ Moreover, recent studies have suggested that *APOE* ε4 may also influence motor symptom progression in PD.^7^ Given the potential influence of *APOE* ε4 on cognition and other nonmotor/motor symptoms in PD, this study aims to investigate the associations between *APOE* ε4 genotypes and motor/nonmotor symptoms in a large sample of patients. In addition, we aimed to investigate the relationship between *APOE* ε4 and cognitive impairment while considering the impact of other factors. By exploring these associations, we aim to enhance our understanding of the potential role of genetic variation in PD symptom development and identify targets for personalized interventions to improve patient outcomes.

## Methods

### Study Design and Fox Insight Database

**FIG. 1:**
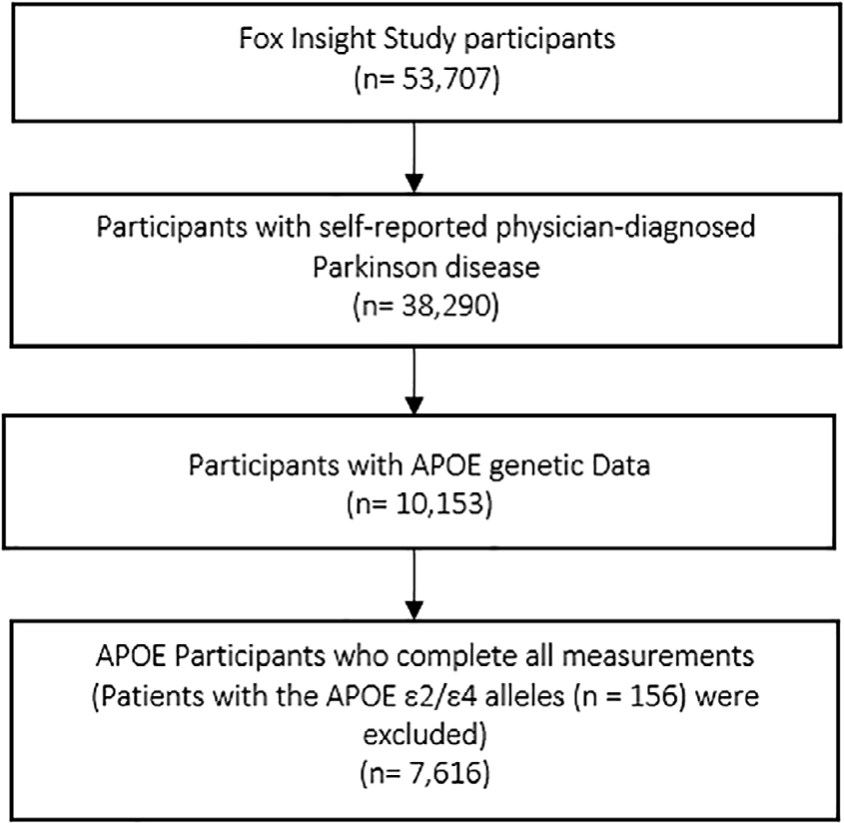
Flowchart of sample collection. *APOE*, apolipoprotein E.

We examined baseline data from participants with PD enrolled in the Fox Insight (FI) study between 2017 and 2020. The FI study, sponsored by The Michael J. Fox Foundation for Parkinson's Research (https://foxinsight-info.michaeljfox.org/insight/explore/insight.jsp), is an ongoing, online clinical trial that is building a large cohort of people with and without PD and capturing self‐reported clinical variables. Study methodology for the FI study has been reported elsewhere in detail.^8^ Briefly, FI study participants are at least 18 years old and were recruited through personal efforts (clinician referrals and events promoting the research) and digital channels (eg, social media advertising, e‐newsletters). Participants complete a standardized set of questionnaires at regular intervals via an online survey platform and receive questionnaires on specific topics (eg, general health, lifestyle, MNMS, socioeconomic situation, quality of life). In this study, we included individuals with PD with *APOE* genetic data and excluded individuals who had incorrectly filled or missing data (see Fig. 1).

### Measures

#### *APOE* Genotyping

For *APOE* genotyping, the 23andMe toolkit was used (DNA was extracted from the saliva of patients). Briefly, *APOE* genotype was genotyped based on 2 single nucleotide polymorphisms (SNPs), rs7412 and rs429358, to distinguish between the *APOE* ε2, ε3, and ε4 alleles. Illumina Infinium Global Screening (Illumina, Mountain View, CA, USA) or OmniExpress‐24 Bead Chip array was used to genotype these SNPs (according to the FI study genetic data manual).^8^ We also excluded patients with the *APOE* ε2/ε4 alleles (n = 156) because the *APOE* ε2 allele, which has potential neuroprotective effects, may have attenuated the harm related to the *APOE* ε4 allele.^9^

#### Clinical Variables

To rate cognitive instrumental activities of daily living (IADL), the Penn Parkinson's Daily Activities Questionnaire (PDAQ‐15) was used. The questionnaire includes 15 items that assess the level of difficulty a participant currently experiences as a result of PD on IADLs such as reading comprehension, medication management, navigation, learning to use new gadgets, financial management/understanding, and orientation. Each question is scored 0 to 4, with a total range of 0 to 60 points. Higher scores indicate better performance on IADLs, and the rating scale is as follows: none = 4, a little = 3, somewhat = 2, a lot = 1, or cannot do = 0. To differentiate between participants with normal/mildly impaired cognitive function and those with severe impairment, a previously validated cut‐off score of <43 was applied. This cut‐off score was validated for this particular assessment conducted by knowledgeable informants and was used in this study because of the lack of a currently validated self‐report measure.^10^, ^11^

Physical activity was assessed with the Physical Activity Scale for the Elderly (PASE). The PASE is a self‐reported questionnaire assessing the duration and frequency of physical activities during the previous week. It consists of the following 3 domains: leisure (5 components), household (6 components), and occupation (1 component). The leisure subscore includes questions about light activity (reading), light recreational activity (bowling, golf), walking; moderate or vigorous physical activity (tennis, swimming), and muscle strengthening (weightlifting). Household activities include light (eg, dusting and washing dishes) and heavy (eg, scrubbing floors and cleaning windows). The occupational activities include questions about hours spent working or volunteering and the amount of physical activity required for work (office work vs. manual labor). However, the occupational data were not published in the FI study because of privacy concerns and therefore have not been included in the PASE calculation. The PASE score was calculated according to the scoring manual and higher scores indicated greater physical activity.^12^

Furthermore, for the assessment of depression, we used the Geriatric Depression Scale 15 (GDS‐15), a short form extracted from the 15 questions of the GDS. The total score is 30 points with validated cutoffs for the short assessment, scores below 5 indicate no depression; 5 to 8, mild symptoms; 9 to 11, moderate symptoms; and 12 to 15, severe symptoms.^13^

For assessing motor functions, the Movement Disorder Society–Sponsored Revision of the Unified Parkinson's Disease Rating Scale (MDS‐UPDRS) Part II was used (Part II consisting of 13 items). All items were scored on a scale from 0 (normal) to 4 (severe), and total scores were obtained from the sum of the corresponding item scores.^14^

The Non‐motor Symptom Questionnaire (NMS) was used to assess nonmotor symptoms. This is a 30‐item, self‐completed, yes/no questionnaire to assess patients' nonmotor symptoms in PD. The items contain 9 domains: gastrointestinal, urinary tract, sexual function, cardiovascular, apathy/attention/memory, hallucinations/delusions, depression/anxiety/anhedonia, sleep/fatigue, pain, and miscellaneous. The positive answers were summed for a total score ranging from 0 to 30. Higher scores indicate worse nonmotor symptom conditions.^15^

The Rapid Eye Movement Sleep Behavior Disorder Single‐Question Screen (RBD1Q) questionnaire is a screening tool used to identify individuals with probable rapid eye movement sleep behavior disorder (RBD). The RBD1Q consists of a single yes/no question that asks, “Have you ever been told, or suspected yourself, that you seem to ‘act out your dreams’ while asleep (eg, punching, flailing your arms in the air, making running movements, etc.)?” This questionnaire has been validated as a useful tool for identifying patients with probable RBD and has been shown to have a high sensitivity and specificity.^16^

Using the Comorbidity Questionnaire, patients were asked about 14 health conditions (high blood pressure, heart disease, asthma, chronic obstructive pulmonary disease, ulcer/stomach disease, diabetes, high blood lipid level, kidney disease, osteoarthritis/degenerative arthritis, rheumatoid arthritis, osteoporosis, cancer disease, depression, other psychiatric diseases). In addition, all “other” reported health problems were asked: (1) whether they have the condition (yes/no), (2) whether they receive treatment for the condition (yes/no), and (3) whether the condition limits their activities (yes/no).^17^

#### Other Variables

The following demographic variables were used: age, sex, income, employment, and educational level, as well as taking PD medication and disease duration.

#### Analysis

Data normality were assessed with the use of Shapiro–Wilk test and histogram analysis. The data were presented as mean, standard deviation (SD), and median with minimum–maximum, and frequencies. Participant baseline characteristics were described according to the *APOE* genotype, ε4 noncarriers (includes ε2/ε2, ε2/ε3, ε3/ε3), *APOE* ε4 heterozygotes (ε3/ε4), and *APOE* ε4 homozygotes (ε4/ε4) and calculated using analysis of variance or Kruskal–Wallis test and the χ^2^ test where appropriate. To explore the associations between different *APOE* genotypes and various motor/nonmotor symptoms, we performed binary logistic regression analysis using *APOE* noncarriers as the reference group. The independent variables included GDS, MDS‐UPDRS Part II, NMS, PDAQ‐15, PASE, and PRBD1Q scores. To adjust for confounding variables, we ran 3 different models: the first adjusted for age and sex; the second additionally adjusted for education, employment, and income; and the final model further adjusted for disease duration, PD medication, and self‐reported comorbidity (as shown in Table 1).

**TABLE 1:** Association between APOE ε4 allele status (APOE ε4 noncarriers vs. heterozygotes and homozygotes) with motor and nonmotor symptoms

Variables	Model 1 OR (95% CI)	*P* value	Model 2 OR (95% CI)	*P* value	Model 3 OR (95% CI)	*P* value
GDS score						
*APOE* noncarriers	Reference		Reference		Reference	
*APOE* ε4 heterozygotes	1.19 (0.99–1.25)	0.127	1.18 (0.97–1.39)	0.156	1.15 (0.92–1.40)	0.172
*APOE* ε4 homozygotes	0.93 (0.67–1.77)	0.672	0.93 (0.63–1.78)	0.715	0.94 (0.62–1.90)	0.746
PDAQ‐15 score						
*APOE* noncarriers	Reference		Reference		Reference	
*APOE* ε4 heterozygotes	0.88 (0.86–0.94)	**0.019**	0.91 (0.86–0.97)	**0.042**	0.92 (0.87–0.99)	**0.049**
*APOE* ε4 homozygotes	1.01 (0.67–1.43)	0.751	1.02 (0.63–1.95)	0.798	1.02 (0.62–2.07)	0.866
MDS‐UPDRS score						
*APOE* noncarriers	Reference		Reference		Reference	
*APOE* ε4 heterozygotes	1.27 (0.97–1.43)	0.132	1.25 (0.96–1.44)	0.174	1.22 (0.92–1.47)	0.205
*APOE* ε4 homozygotes	1.05 (0.97–1.07)	0.751	1.05 (0.96–1.11)	0.788	1.04 (0.94–1.12)	0.795
NMS score						
*APOE* noncarriers	Reference		Reference		Reference	
*APOE* ε4 heterozygotes	1.13 (0.84–1.17)	0.302	1.12 (0.86–1.18)	0.341	1.09 (0.85–1.21)	0.388
*APOE* ε4 homozygotes	1.04 (0.95–1.15)	0.772	1.03 (0.93–1.12)	0.779	1.02 (0.92–1.09)	0.796
PASE score						
*APOE* noncarriers	Reference		Reference		Reference	
*APOE* ε4 heterozygotes	0.92 (0.91–1.17)	0.456	0.93 (0.91–1.16)	0.481	0.94 (0.91–1.19)	0.532
*APOE* ε4 homozygotes	1.12 (0.97–1.15)	0.271	1.12 (0.95–1.19)	0.279	1.11 (0.91–1.25)	0.398
PRBD1Q (yes)						
*APOE* noncarriers	Reference		Reference		Reference	
*APOE* ε4 heterozygotes	1.19 (0.95–1.29)	0.452	1.18 (0.96–1.29)	0.474	1.17 (0.95–1.28)	0.446
*APOE* ε4 homozygotes	0.80 (0.45–1.08)	0.287	0.81 (0.45–1.10)	0.281	0.79 (0.51–1.18)	0.240

*Note*: Model 1, adjusted for age and sex; Model 2, adjusted for age, sex, education, employment, and income; Model 3, adjusted for age, sex, education, employment, income, disease duration, and Parkinson's disease medication and self‐reported comorbidity. *APOE* ε4 noncarriers include ε2/ε2, ε2/ε3, and ε3/ε3; *APOE* ε4 heterozygotes include ε3/ε4; and *APOE* ε4 homozygotes include ε4/ε4. Bolded *P* values indicate statistical significance at the *P* < 0.05 level.

Abbreviations: *APOE*, apolipoprotein E; OR, odds ratio; CI, confidence interval; GDS, Geriatric Depression Scale; PDAQ‐15, Penn Parkinson's Daily Activities Questionnaire; MDS‐UPDRS, Movement Disorder Society–Sponsored Revision of the Unified Parkinson's Disease Rating Scale; NMS, Non‐motor Symptom Questionnaire; PASE, Physical Activity Scale for the Elderly; PRBD1Q, Probably REM Sleep Behavior Disorder Single Question; REM, rapid eye movement.

To assess the associations between cognitive IADL and GDS, NMS, MDS‐UPDRS Part II, PASE, and PRBD1Q scores and *APOE* genotypes, we conducted both univariate and multivariate logistic regression analyses (as shown in Table 2). For this purpose, we dichotomized the cognitive IADL variable (PDAQ‐15) based on a cutoff score of 43.^10^ In the multivariate analysis, we included all significant independent variables from the univariate analysis along with additional covariates such as age, sex, education, employment, income, disease duration, PD medication use, and comorbidity in the model. A *P* value less than 0.05 was considered statistically significant. We performed all analyses using the statistical software package SPSS 28.0 (IBM Corp, Armonk, NY). All calculations were considered exploratory, and we did not adjust for type II error.

**TABLE 2:** Association of cognitive impairment in instrumental activities of daily living with motor/nonmotor symptoms and APOE genotypes in Parkinson's disease

Variables	Univariate OR (95% CI)	*P* value	Multivariate AOR (95% CI)	*P* value
PDAQ‐15 score cutoff <43 vs. ≥43				
GDS score	1.58 (1.50–1.63)	<0.001	1.28 (1.23–1.34)	<0.001
MDS‐UPDRS Part II score	1.17 (1.12–1.19)	<0.001	1.07 (1.06–1.09)	<0.001
NMS score	1.35 (1.27–1.39)	<0.001	1.22 (1.19–1.25)	<0.001
PASE score	0.98 (0.97–0.99)	<0.001	0.99 (0.99–1.00)	0.009
PRBD1Q	1.68 (1.22–2.01)	<0.001	1.05 (1.23–1.26)	0.376
*APOE* ε4 heterozygotes^a^	1.23 (1.03–1.47)	0.038	1.23 (0.97–1.54)	0.060
*APOE* ε4 homozygotes^a^	1.21 (0.75–2.02)	0.425		

*Note*: AOR for age, sex, education, employment, income, disease duration, taking Parkinson's disease medication, and comorbidity. PDAQ‐15 score cutoff <43 vs. ≥43.

Abbreviations: *APOE*, apolipoprotein E; OR, odds ratio; CI, confidence interval; AOR, adjusted odds ratio; PDAQ‐15, Penn Parkinson's Daily Activities Questionnaire; GDS, Geriatric Depression Scale; MDS‐UPDRS, Movement Disorder Society–Sponsored Revision of the Unified Parkinson's Disease Rating Scale; NMS, Non‐motor Symptom Questionnaire; PASE, Physical Activity Scale for the Elderly; PRBD1Q, Probably REM Sleep Behavior Disorder Single Question; REM, rapid eye movement.

^a^ Reference *APOE* ε4 noncarriers.

## Results

The sample selection of our cohort is outlined in Figure 1. From the 53,707 participants of the FI study, we included those with available *APOE* genotype data and complete data, yielding a final sample of 7616 individuals with a mean age 65.3 (SD 9.2) years and a slightly lower proportion of female participants (43.9%). With respect to *APOE* genotype, 78.7% were noncarriers of the ε4 allele, 19.9% were *APOE* ε4 heterozygotes, and 1.4% were ε4 homozygotes. Detailed information on participants' characteristics can be found in Table 3. No significant differences were found between *APOE* ε4 genotypes in all reported variables: age, sex, disease duration, PD medication, education, employment, income, comorbidity score, depression, cognition and daily activities, movement experiences, nonmotor symptoms, physical activities, and PRSBD (Table 3).

**TABLE 3:** Characteristics of the online participants and differences in the APOE ε4 genotypes

Variables	Total sample, N = 7616	*APOE* ε4 noncarriers, n = 5992	*APOE* ε4 heterozygotes, n = 1517	*APOE* ε4 homozygotes, n = 107	*P* value
Age (years) mean (SD)	65.3 (9.6)	65.4 (9.4)	65.0 (9.7)	64.8 (10.4)	0.544^a^
Sex					
Female	3329 (43.7)	2610 (43.6)	672 (44.3)	47 (43.9)	0.941^b^
Disease duration					
<2 years	3568 (46.8)	2792 (46.6)	726 (47.9)	50 (46.7)	0.617^b^
2–10 years	3331 (43.7)	2642 (44.1)	645 (42.5)	44 (41.1)	
>10 years	717 (9.4)	558 (9.3)	146 (9.6)	13 (12.1)	
PD medication (yes)	6868 (90.2)	5397 (90.1)	1374 (90.6)	97 (90.7)	0.531^b^
Education					
High school	537 (7.1)	431 (7.2)	97 (6.4)	9 (8.4)	0.708^b^
Some college	1245 (16.3)	993 (16.6)	235 (15.5)	17 (15.9)	
College degree	638 (8.4)	487 (8.1)	144 (9.5)	7 (6.5)	
Advanced degree	5190 (68.1)	4077 (68.0)	1039 (68.5)	74 (69.2)	
Prefer not to answer	6 (0.1)	4 (0.1)	2 (0.1)	0	
Employment					
Full‐time	1679 (22.1)	1306 (21.8)	347 (22.9)	26 (24.3)	0.515^b^
Part‐time	579 (7.6)	449 (7.5)	123 (8.1	7 (6.5)	
Retired	4857 (63.8)	3849 (64.2)	940 (61.9)	68 (63.6)	
Unemployed	474 (6.2)	369 (6.2)	99 (6.5)	6 (5.6)	
Prefer not to answer	27 (0.4)	19 (0.3)	8 (0.5)	0	
Income (dollars)					
<$34,999	965 (12.7)	775 (12.9)	177 (11.7)	13 (12.1)	0.374^b^
$35,000–$74,999	1962 (25.8)	1557 (26.0)	380 (25.1)	25 (23.4)	
$75,000–$99,999	1176 (15.4)	909 (15.2)	244 (16.1)	23 (21.5)	
>$100,000	2709 (35.6)	2124 (35.4)	555 (36.3)	30 (28.0)	
Prefer not to answer	804 (10.6)	627 (10.5)	161 (10.6)	16 (15.0)	
Comorbidity score (CQ), median (min‐max)	4 (0–23)	4 (0–23)	4 (0–17)	4 (0–14)	0.771^c^
Depression (GDS 15 score), median (min–max)	6 (1–14)	6 (1–14)	6 (1–13)	5 (1–11)	0.289^c^
Normal (GDS <5)	1312 (17.2)	1034 (17.3)	256 (16.9)	22 (20.6)	0.852^b^
Mild (GDS 5–8)	5442 (71.5)	4284 (71.3)	1085 (71.5)	73 (68.2)	
Moderate (GDS 9–11)	804 (10.6)	629 (10.5)	163 (10.7)	12 (11.2)	
Severe (GDS 12–15)	58 (0.8)	45 (0.8)	13 (0.9)	0	
Cognition and daily activities	54 (1–60)	54 (1–60)	51 (3–56)	53 (1–60)	0.104^c^
(PDAQ‐15 score), median (min‐max)					
Normal/mildly (cutoff ≥43)	6276 (82.4)	4911 (82.0)	1276 (84.1)	89 (83.2)	0.143^b^
Severe (cutoff <43)	1340 (17.6)	1081 (18.0)	241 (15.9)	18 (16.8)	
Movement experiences (MDS‐UPDRS Part II score), median (min‐max)	9 (0–51)	10 (0–51)	9 (0–44)	9 (0–47)	0.559^c^
Nonmotor symptoms	10 (1–30)	10 (1–30)	10 (1–28)	10 (2–22)	0.575^c^
NMS, median (min‐max)					
Physical activities (PASE)	113.7 (0–617)	113.8 (0–617)	113.5 (0–534)	116.4 (0–355)	0.876^c^
Median (min–max)					
Leisure activities	106 (0–351)	106 (0–351)	106 (0–351)	124 (0–305)	0.177^c^
Household activities	80 (0–170)	80 (0–171)	80 (0–171)	80 (0–171)	0.916^c^
Probably REM sleep behavior disorder (PRBD1Q)	3157 (41.5)	2504 (41.8)	614 (40.5)	39 (36.4)	0.352^b^

*Note*: Values are n (%) unless otherwise noted. *APOE* ε4 noncarriers include ε2/ε2, ε2/ε3, and ε3/ε3; *APOE* ε4 heterozygotes include ε3/ε4; and *APOE* ε4 homozygotes include ε4/ε4.

Abbreviations: APOE, apolipoprotein E; SD, standard deviation; PD, Parkinson's disease; CQ, Comorbidity Questionnaire; max, maximum; min, minimum; GDS, Geriatric Depression Scale; PDAQ‐15, Penn Parkinson's Daily Activities Questionnaire; MDS‐UPDRS, Movement Disorder Society–Sponsored Revision of the Unified Parkinson's Disease Rating Scale; NMS, Non‐motor Symptom Questionnaire; PASE, Physical Activity Scale for the Elderly; RBD1Q, REM Sleep Behavior Disorder Single Question; REM, rapid eye movement.

^a^ One‐way analysis of variance in metric data.

^b^ χ^2^ test in categorical data.

^c^ Kruskal‐Wallis test.

In Table 1, the fully adjusted model (model 3) revealed that individuals with *APOE* ε4 heterozygosity had an 8% lower odds (odds ratio [OR], 0.92; 95% confidence interval [CI], 0.87–0.99) of having a higher PDAQ‐15 score (a higher score reflects better cognition) than those without the allele. However, there was no significant association between *APOE* ε4 homozygosity and PDAQ‐15 across all 3 models. Furthermore, none of the other independent variables, including GDS, NMS, PASE, MDS‐UPDRS Part II, and PRBD1Q scores, were significantly linked to *APOE* ε4 heterozygosity or homozygosity in any of the models.

Table 2 reveals significant predictors of cognitive impairment in the IADL of patients with PD. Univariate analysis pointed to all variables, except *APOE* ε4 homozygotes, as significant. However, multivariate analysis refined these predictors to GDS, NMS, MDS‐UPDRS Part II, and PASE scores. Each 1‐point increment in GDS, NMS, and MDS‐UPDRS Part II score raised the cognitive impairment odds by 28%, 22%, and 7%, respectively. Conversely, higher PASE scores, indicating increased physical activity, lowered cognitive impairment risk. *APOE* ε4 heterozygotes showed no significant effect in the multivariate analysis.

## Discussion

This is one of the largest data sets of individuals that describes the genetic variability of *APOE* ε4 genotype associated with motor and nonmotor symptoms in PD. In the present study, we found a modest but significant association at least in *APOE* ε4 heterozygotes with disabilities in cognitive IADL status. The fact that the association remained significant even after an adjustment for several cofounders indicates a marginal detrimental impact of *APOE* ε4 on cognition in IADL of individuals with PD. However, we did not have enough statistical power to detect the effect of the ε4 allele when ε4 homozygote alleles were compared with *APOE* ε4 noncarriers. Although several studies have demonstrated an overrepresentation of *APOE* ε4 carriers among individuals with PD cognitive impairment and dementia, others have been equivocal or provided only modest evidence.^18^, ^19^, ^20^ It is important to note that many existing studies do not differentiate between ε4 homozygotes and heterozygotes. This may contribute to the inconsistency observed in their results. Further various methodological factors could account for these discrepancies, such as differing approaches to assessing cognitive decline, limited sample sizes, and varied characteristics of the study populations, including differences in age at disease onset. To gain a more precise understanding of the role of this gene in cognitive impairments associated with PD, future research should aim to more clearly distinguish between ε4 homozygotes and heterozygotes. Despite these inconsistencies, the evolving evidence suggests that the *APOE* ε4 allele could be a risk factor for cognitive decline in neurodegenerative diseases. However, the underlying pathophysiological mechanisms remain to be fully elucidated.^5^, ^21^

The *APOE* ε4 allele is widely recognized in scientific literature for its role in accelerating cognitive and motor decline in PD.^5^, ^22^, ^23^ Our study indicates several significant predictors for cognitive impairments in the IADL of patients with PD. In the univariate analysis, all variables proved significant except for *APOE* ε4 homozygotes. In the multivariate analysis, these predictors were refined to the GDS, NMS, MDS‐UPDRS Part II, and PASE scores. *APOE* ε4 heterozygotes did not show a significant effect in the multivariate analysis. *APOE* ε4 is associated with the accumulation and deposition of amyloid‐β (Aβ) peptides, a hallmark of neurodegeneration. The interaction of *APOE* ε4 with the low‐density lipoprotein receptor‐related protein 1 could exacerbate the Aβ pathology, pointing toward a possible mechanism for the *APOE* ε4–induced cognitive decline.^24^ However, our results suggest that the influence of *APOE* ε4 on cognitive impairment may be more nuanced and possibly modulated by other factors such as depression and physical activity. Although the *APOE* ε4 allele is linked with an increased risk for depression and cognitive impairments,^25^ lifestyle factors such as physical activity and a resultant potential better motor condition appear to be associated with a better cognitive state.^26^, ^27^ This suggests the possibility that lifestyle changes could offset genetic predispositions in patients with PD. However, to fully understand these interactions, further investigation into the role of *APOE* ε4, depression, physical activity, and cognition in the context of PD is warranted.

Our study did not find an association between the *APOE* ε4 allele and motor or nonmotor symptoms in PD individuals based on the patient‐reported MDS‐UPDRS Part II data. However, the broader literature provides a nuanced perspective. Some studies using the clinician‐rated Part III of the MDS‐UPDRS have suggested an association between the *APOE* ε4 allele and risk of motor symptoms,^7^, ^27^ whereas another study found no such association.^28^ It is plausible that the lack of association in our study could be attributed to the potential discrepancy between self‐reported and clinician‐assessed symptoms, leading to different results.^29^, ^30^ One specific example of this complexity is illustrated by the work of Pu et al,^7^ who investigated the relationship between the *APOE* ε4 genotype and the progression of motor symptoms in PD. Their findings suggest that the impact of the *APOE* ε4 allele might be more pronounced in the neocortex, where AD and Lewy body disease pathologies are found, rather than in the subcortical and brainstem structures where Lewy bodies are predominantly found in PD. Specifically, their study indicated that *APOE* ε4 was associated with the progression of motor symptoms during the middle or late stages of PD, especially in patients who were either Aβ positive or RBD negative. The authors also posited that *APOE* ε4 might facilitate the propagation of extracellular α‐synuclein in cortical regions during the mid‐to‐late stages of the disease, suggesting a potential direct interaction between *APOE* ε4 and α‐synuclein. These findings underscore the potential role of *APOE* ε4 in the propagation of α‐synuclein pathology in PD, highlighting the intricate interplay between genetic and pathological factors in the progression of the disease.

Given that longitudinal studies represent a gold standard for tracking disease progression but are traditionally hampered by small sample size, the power of this study is significantly enhanced through its size. In this study, we were able to significantly enhance the power of our analysis by examining *APOE* variants in a large cohort of 7616 individuals.

### Limitations

The present study has several limitations: First, as a result of the cross‐sectional design of the study, conclusions are limited to a specific point in time. Second, although online surveys have the advantage of collecting data from many individuals in a short period of time, the major limitation is that all results are based on self‐report. In addition, the PD diagnosis was self‐reported without independent review by a movement disorder specialist, which limits the possibility of ruling out that some patients may actually have a form of atypical parkinsonism that may respond differently to cognitive performance in IADL than PD. Therefore, the lack of quantification of the different measures should be considered when interpreting the results. Furthermore, although the PDAQ‐15 measures the potential impact of cognitive IADL and is correlated with cognition, cognition was not directly assessed in this study and was solely reliant on self‐reported data. Studies have revealed that individuals tend to underestimate their cognitive impairments in self‐report questionnaires.^31^ Physical activity were quantified with the self‐reported PASE questionnaire, which has the risk of introducing recall bias, although the PASE is strongly correlated with objective measures of physical activity. As such, other objective measurements (eg, fitness trackers with global positioning system capability) should be considered in the future to provide more precise and accurate estimates of physical activity and to reduce measurement errors that are related to these issues. However, such measures are often time‐consuming, expensive, and also challenging in terms of data protection, and such barriers to feasibility mean that these measures are unlikely to be implemented in online surveys.

It is important to note that individuals with more severe cognitive impairments may have been unable to participate or complete the questionnaire fully, which may have led to an underrepresentation of severe cognitive impairment, potentially limiting the generalizability of the findings. However, compared with academic centers where response rates are traditionally low, the FI study was able to achieve a high response rate and obtain a large sample size, which allows obtaining a comprehensive understanding of people with PD. This online approach can improve accessibility for individuals with mobility or cognitive impairments and could be an effective strategy for future research on PD and other neurological disorders, especially when attempting to recruit underrepresented populations.

## Conclusion

Our study found that *APOE* ε4 genotype (heterozygote) is modestly associated with cognitive impairment in IADL among individuals with PD. However, no significant associations were observed between *APOE* ε4 and motor or other nonmotor symptoms in PD. Although a link between cognition and *APOE* ε4 exists in patients with PD, our results suggest that *APOE* ε4 plays a secondary role when considering multiple factors such as depression, physical activity, and other nonmotor symptoms, which have a stronger impact on cognitive impairment. Further longitudinal studies are necessary to better understand the role of *APOE* ε4 genotype in cognitive impairment and to develop specific prevention strategies for individuals at high genetic risk.

## Author Roles

(1) Research Project: A. Conception, B. Organization, C. Execution; (2) Statistical Analysis: A. Design, B. Execution, C. Review and Critique; (3) Manuscript Preparation: A. Writing of the First Draft, B. Review and Critique.

A.K.: 1A, 1B, 1C, 2A, 2B, 3A

S.H.: 1A, 3B

M.W.: 2B, 2C, 3B

J.S.: 1A, 1B, 1C, 2C, 3B

## Disclosures

**Ethical Compliance Statement:** The authors confirm that informed patient consent was not required for this work. We confirm that we have read the journal's position on issues involved in ethical publication and affirm that this work is consistent with those guidelines. The authors confirm that the approval of an institutional review board was not required for this work.

**Funding Sources and Conflicts of Interest:** This research did not receive any specific grant from funding agencies in the public, commercial, or not‐for‐profit sectors. There are no relevant conflicts of interest to disclose.

**Financial Disclosures for the Previous 12 Months:** The authors declare that there are no additional disclosures to report.
